# WIP Modulates Oxidative Stress through NRF2/KEAP1 in Glioblastoma Cells

**DOI:** 10.3390/antiox9090773

**Published:** 2020-08-20

**Authors:** Maribel Escoll, Diego Lastra, Natalia Robledinos-Antón, Francisco Wandosell, Inés María Antón, Antonio Cuadrado

**Affiliations:** 1 Department of Biochemistry, Medical College, Autonomous University of Madrid (UAM), Arzobispo Morcillo 4, 28029 Madrid, Spain; mescoll@iib.uam.es (M.E.); diegolastra@iib.uam.es (D.L.); nrobledinos@iib.uam.es (N.R.-A.); 2 Instituto de Investigaciones Biomédicas Alberto Sols (CSIC-UAM), Arturo Duperier 4, 28029 Madrid, Spain; 3 Instituto de Investigación Sanitaria La Paz (IdiPaz), Pedro Rico 6, 28029 Madrid, Spain; 4 Centro de Investigación Biomédica en Red de Enfermedades Neurodegenerativas (CIBERNED), Valderrebollo 5, 28049 Madrid, Spain; fwandosell@cbm.csic.es (F.W.); ianton@cnb.csic.es (I.M.A.); 5 Centro de Biología Molecular Severo Ochoa (CSIC-UAM), Universidad Autónoma Madrid, Nicolás Cabrera 1, 28049 Madrid, Spain; 6 Centro Nacional de Biotecnología (CNB-CSIC), Department of Cellular and Molecular Biology, Darwin 3, 28049 Madrid, Spain

**Keywords:** oxidative stress, redox, antioxidants, cytoskeleton

## Abstract

Due to their high metabolic rate, tumor cells produce exacerbated levels of reactive oxygen species that need to be under control. Wiskott–Aldrich syndrome protein (WASP)-interacting protein (WIP) is a scaffold protein with multiple yet poorly understood functions that participates in tumor progression and promotes cancer cell survival. However, its participation in the control of oxidative stress has not been addressed yet. We show that WIP depletion increases the levels of reactive oxygen species and reduces the levels of transcription factor NRF2, the master regulator of redox homeostasis. We found that WIP stabilizes NRF2 by restraining the activity of its main NRF2 repressor, the E3 ligase adapter KEAP1, because the overexpression of a NRF2^ΔETGE^ mutant that is resistant to targeted proteasome degradation by KEAP1 or the knock-down of KEAP1 maintains NRF2 levels in the absence of WIP. Mechanistically, we show that the increased KEAP1 activity in WIP-depleted cells is not due to the protection of KEAP1 from autophagic degradation, but is dependent on the organization of the Actin cytoskeleton, probably through binding between KEAP1 and F-Actin. Our study provides a new role of WIP in maintaining the oxidant tolerance of cancer cells that may have therapeutic implications.

## 1. Introduction

Cancer cells undergo modifications in morphology associated with the cytoskeleton that lead to changes in the cell shape, adhesion, and contractility. These changes favor migratory capacity, anchorage-independent growth, and the capacity to metastasize [1,2]. Actin filaments play an essential role in cytoskeletal formation and remodeling. Actin-rich adhesions establish close contact with the substratum and are structural components of the extracellular matrix degradative structures, podosomes and invadopodia [3]. At this time, the mechanisms involved in the remodeling of the Actin cytoskeleton are still very little defined in these structures. However, it is known that the Actin-related protein Wiskott–Aldrich syndrome protein (WASP)-interacting protein (WIP), among other proteins [4,5], plays a fundamental role.

Human WIP is a proline-rich, ubiquitously expressed scaffold protein with multiple, yet poorly understood, functions in cell signaling, endocytosis, and cytoskeleton remodeling [5,6]. WIP participates in the formation of Actin-rich structures, such as immune synapses, filopodia, lamellipodia, stress fibers, and podosomes [5]. WIP-deficient mice exhibit a reduced formation of podosomes [7,8,9] and invadopodia [5,9], evidencing its relevance in the regulation of Actin polymerization. All these observations also support the pro-tumoral role of WIP by endowing some cancer cells with anchorage-independent growth and higher motility [6,10]. In fact, a high WIP expression correlates with poor prognosis in patients with pancreatic ductal adenocarcinoma [11], lung cancer [12], colorectal cancers, breast cancer, and gliomas [13].

WIP also increases cell survival and the proliferation of cancer cells by yet poorly defined mechanisms [6]. Its participation in redox control, crucial for the survival of cancer cells, has not been addressed yet. The highly proliferative activity of cancer cells leads to increased ROS levels and changes in the redox metabolism that favor protumoral functions such as genetic instability, cell migration, and mitotic signaling [14]. Loss of cell contact inhibition, survival upon matrix detachment, migratory ability, and tissue infiltration are all critical features of tumor metastasis, and ROS have been implicated in these processes [15,16]. However, the sustained elevation of ROS levels induces cell cycle arrest, senescence, and cancer cell death [17,18,19]. Therefore, tumor cells develop strategies to restrain ROS production to tolerable levels by activating endogenous antioxidant systems such transcription factor NRF2, the master regulator of redox metabolism [20,21], as well as many other cytoprotective functions related to metabolic reprogramming that contribute to cell proliferation in cooperation with other oncogenic pathways [22,23].

In this study, we found that, in the absence of WIP, the NRF2 levels are reduced due to its increased ubiquitin-proteasome degradation mediated by the E3 ligase adapter KEAP1. Our study provides a new role of WIP in maintaining the oxidant tolerance of cancer cells.

## 2. Materials and Methods

### 2.1. Cell Culture and Reagents

The validated cell lines HEK293T (ATCC, CRL-11268), U-373 MG (ATCC, HTB-17), and U-87 MG (ATCC, HTB-14) were maintained in Dulbecco’s Modified Eagle Medium supplemented with 10% fetal bovine serum in 5% CO_2_ at 37 °C conditions. Sulforaphane (SFN), MG132, leupeptin, and ammonium chloride were purchased from Sigma-Aldrich. The gene IDs of this study include: *WIP/WIPF1* (WASP-interacting protein; gene ID: 7456), *NFE2L2/NRF2* (Nuclear factor erythroid 2-related factor 2; gene ID: 4780), *KEAP1* (kelch-like ECH-associated protein 1; gene ID: 9817), *HMOX1* (heme oxygenase-1; gene ID: 3162), *NQO1* (NAD(P)H quinone dehydrogenase 1; gene ID: 1728), and *GCLM* (glutamate-cysteine ligase modifier subunit; gene ID: 2730).

### 2.2. Immunoblotting

This protocol was performed as described in [24]. Briefly, the cells were homogenized in lysis buffer (TRIS pH 7.6 50 mM, 400 mM NaCl, 1 mM EDTA, 1 mM EGTA, and 1% SDS) and the samples were heated at 95 °C for 15 min, sonicated, and pre-cleared by centrifugation. The proteins were resolved in SDS-PAGE and transferred to Immobilon-P (Millipore) membranes. The proteins of interest were detected with the following primary antibodies: Actin (sc-1616, Santa Cruz Biotechnology, Dallas, TX, USA), GAPDH (Glyceraldehyde 3-phosphate dehydrogenase; CB1001, Merck Millipore, Burlington, MA, USA), KEAP1 (8047, Cell Signaling Techonology, Danvers, MA, USA), LaminB (sc-6217, Santa Cruz Biotechnology), LC3 (2775, Cell Signaling Technology), NRF2 (homemade and validated in [25]), NQO1 (ab2346, Abcam, Cambridge, UK), p62 (P0067-200UL, Sigma Aldrich, St. Louis, MO, USA), and WIP (sc-25533, Santa Cruz Biotechnology). Proper peroxidase-conjugated secondary antibodies were used for detection by enhanced chemiluminescence (GE Healthcare, Chicago, IL, USA).

### 2.3. Lentiviral Vector Production and Infection

Pseudotyped lentiviral vectors were produced in HEK293T cells transiently co-transfected with the corresponding lentiviral vector (10 µg), the packaging plasmid pSPAX2 (6 µg; Addgene code 12260, Watertown, MA, USA), and theplasmid pMD2G, expressing the glycoprotein of the vesicular stomatitis virus (6 µg; Addgene code 12259), using the Lipofectamine Plus reagent according to the manufacturer’s instructions (Invitrogen). Lentiviral vector shRNA control (shco) (Addgene code 1864), shWIP (NM_ 003387 TRCN0000029825), and shKEAP1 (NM_ 012289 TRCN0000154657) were purchased from Sigma-Aldrich (MISSION shRNA). The lentiviral vectors pWPXL-NRF2^WT^, NRF2^ΔETGE^, and NRF2^6SA^ were homemade from previous pcDNA3 constructs using the expression vector pWPXL (Addgene, 12257). The cells were infected in the presence of 4 μg/mL of polybrene (Sigma-Aldrich) and selected with 1 μg/mL of puromycin (Sigma-Aldrich).

### 2.4. Analysis of mRNA Levels

The total RNA extraction and qRT-PCR were performed as detailed in [26]. Primer sequences are shown in Supplementary Table S1. Data analysis was based on the ∆∆CT method, with the normalization of the raw data by the geometric mean to the housekeeping genes *GAPDH* and *TBP* (Applied Biosystems, Foster City, CA, USA). All the PCRs were performed in triplicate.

### 2.5. Immunofluorescence and Image Analysis

The cells were adhered on coverslips and fixed with 4% paraformaldehyde. Immunofluorescence was performed as described in [27]. Briefly, the cells were washed, blocked, and permeabilized in PBS containing 0.5% Triton X-100% and 3% bovine serum albumin and incubated for 16 h at 4 °C. Then, the cells were incubated for 2 h with the HA primary antibodies (1:400) (MMS-101R, Covance, Princeton, NJ, USA) and then for 45 min with Alexa Fluor546-conjugated Phalloidin (1:200) (A22283, Thermo Fisher Scientific, Waltham, MA, USA), DAPI (1:1000) (D1306, Thermo Fisher Scientific), and secondary antibodies coupled to Alexa Fluor 488 (1:500) (Life Technologies-Molecular Probes, Grand Island, NY, USA). Images were quantified using the Fiji Software (http://fiji.sc/Fiji). The fluorescence intensity and M1 and M2 colocalization coefficients were measured by Manders analysis using the JACoP plug-in. The co-localization coefficients, M1 and M2, are proportional to the amount of fluorescence of the co-localizing objects in each component of the image, relative to the total fluorescence in that component [28].

### 2.6. Flow Cytometry Determination of Reactive Oxygen Species

Intracellular reactive oxygen species (ROS) were detected in a FACScan flow cytometer (Becton-Dickinson) with hydroethidine (HE) (Thermo Fisher Scientific), which, upon oxidation, emits orange fluorescence (Excitation/Emission: 518/606 nm). The cells were incubated for 1 h at 37 °C with 2 μM of HE and then detached from the plate, washed once with cold PBS, and analyzed immediately.

### 2.7. Statistical Analyses

Data are presented as mean ± S.D. (standard deviation) or S.E.M. (standard error of the mean), as indicated in each case. Statistical assessments of the differences between groups were analyzed using the GraphPad Prism 5 software by an unpaired Student’s *t*-test or one-way ANOVA, as indicated in the legends to the figures.

## 3. Results

### 3.1. WIP Knocked-Down Cells Exhibit Increased ROS and Decreased NRF2 Levels

**Figure 1:**
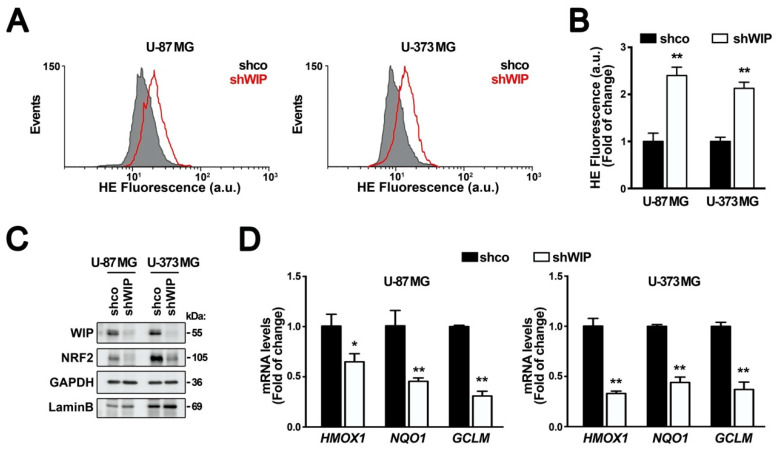
WIP knocked-down cells exhibit increased evels of reactive oxygen species (ROS) and decreased NRF2. U-87 MG and U-373 MG glioblastoma cells were transduced with lentiviral vectors containing shcontrol (shco) or human shWIP. (**A**) Changes in intracellular ROS were determined by HE staining (a.u., arbitrary units of fluorescence emission) (*n* = 3). (**B**) Flow cytometry analysis of shNRF2-induced intracellular ROS production in HE stained cells (a.u., arbitrary units of fluorescence emission). A representative sample of 10,000 cells is shown for each condition. Data are presented as mean ± S.D. ** *p* ≤ 0.01 according to a Student’s *t*-test. (**C**) Representative immunoblots of WIP, NRF2, GAPDH, and LaminB as loading controls (*n* = 3). (**D**) Messenger RNA (mRNA) levels of *HMOX1*, *NQO1*, *GCLM* were determined by qRT-PCR and normalized by the geometric mean of *GAPDH* and *TBP*. Data are presented as mean ± S.D. (*n* = 3) * *p* ≤ 0.05, ** *p* ≤ 0.01 according to a Student’s *t*-test.

First, we determined if WIP can generate changes in the basal redox state of tumor cells. We silenced WIP in the glioblastoma cell lines U-87 MG and U-373 MG with a lentiviral vector expressing shWIP (Supplemental Figure S1). After three days of interference, the intracellular ROS status was analyzed using the superoxide-sensitive fluorescent probe hydroethidine (HE). In both cell lines, the WIP knock-down led to an increase in the ROS levels (Figure 1A,B). Considering that transcription factor NRF2 is a master regulator of genes involved in redox metabolism, we analyzed possible changes in the levels of this protein. We observed that WIP-depleted glioblastoma cells presented low NRF2 levels compared to the transduced cells with the control vector (Figure 1C and Supplemental Figure S1). Moreover, the decrease in NRF2 correlated with reduced transcriptional activity, reflected as the lower mRNA levels of three target genes, *HMOX1*, *NQO1*, and *GCLM*, encoding heme oxygenase-1, NADPH quinone oxidoreductase, and modulator γ-glutamyl cysteine ligase, respectively (Figure 1D).

### 3.2. The Regulation of NRF2 by WIP is KEAP1-Dependent

**Figure 2:**
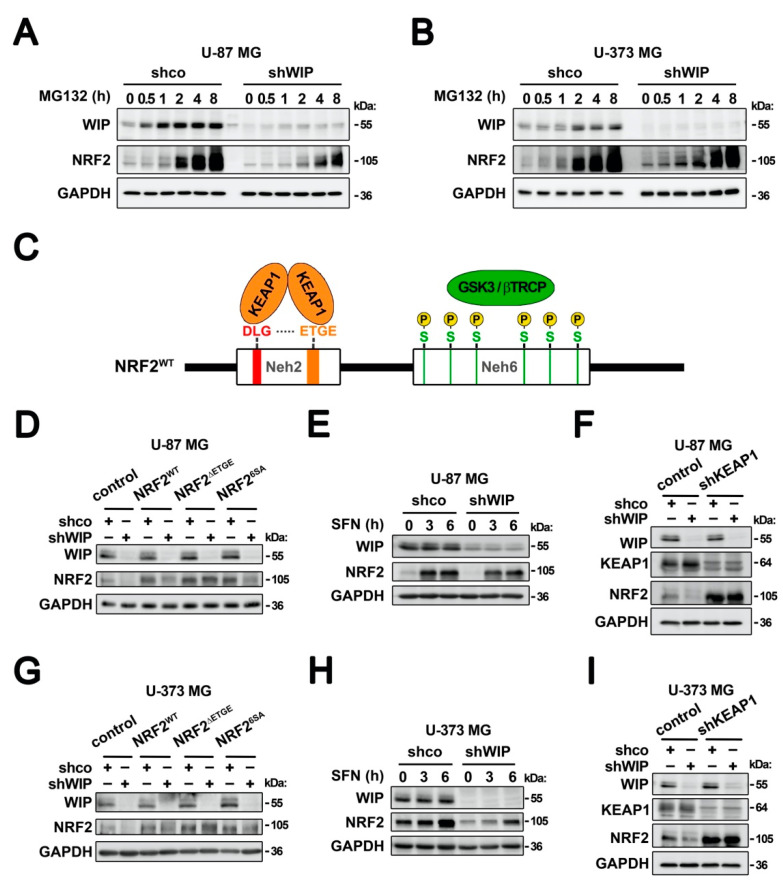
The regulation of NRF2 by WIP is KEAP1-dependent. (**A**,**B**) Time-dependent accumulation of NRF2 in control and WIP-silenced U-87 MG (**A**) and U-373 cells (**B**) and cells treated with 10 µM of MG132. (**C**) Scheme of the two domains of NRF2, Neh2 and Nhe6, which are targeted for proteasome degradation. A KEAP1 homodimer binds the N-terminal region of NRF2 (Neh2 domain) through interaction with a low affinity (DLG) and high affinity (ETGE) amino acid motifs. Thus, the mutated version of NRF2 that lacks the ETGE motif (NRF2^ΔETGE^) is insensitive to KEAP1-mediated degradation [20]. On the other hand, several phosphorylation events, in which Glycogen synthase kinase 3 (GSK-3) is an essential kinase, generate a degradation domain (phosphodegron) that is recognized by the E3 ligase adapter β-TrCP. Thus, the mutated version of NRF2 in which six serines involved in the generation of the phosphorylation have been changed to alanines (NRF2^6SA^) generates a NRF2 insensitive to β-TrCP-mediated degradation [29]. (**D**,**G**) Immunoblots of WIP, NRF2, and GAPDH as a loading control of U-87 MG **(D)** and U-373 MG (**G**) cells that were transduced with lentiviral vectors expressing NRF2^WT^, NRF2^ΔETGE^, NRF2^6SA^ or with empty vector as control and then with a lentivirus expressing shco or shWIP. (**E**,**H**) immunoblots of WIP, NRF2, and GAPDH as a loading control of U-87 MG (**E**) and U-373 MG (**H**) glioblastoma cells that were transduced with lentiviral vectors containing shco or shWIP and then treated with 15 µM of SFN. (**F**,**I**) Immunoblots of WIP, KEAP1, NRF2, and GAPDH as a loading control of U-87 MG (**F**) and U-373 MG (**I**) glioblastoma cells that were transduced with lentiviral vectors containing shco or shKEAP1 or shWIP, as indicated.

We submitted control and WIP-silenced U-87 MG and U-373 MG cells to the proteasome inhibitor MG132 (Figure 2A,B). In the control cells, both WIP and NRF2 accumulated progressively in the presence of the proteasome inhibitor, indicating that both proteins are turned over by this mechanism. However, in the WIP-silenced cells, MG132 still accumulated NRF2. These observations indicate that WIP-depletion leads to the exacerbated degradation of NRF2 by the proteasome, as MG132 can rescue this effect.

NRF2 is a short half-life protein with exquisite post-translational regulation by at least two different E3 ligase adapters, KEAP1 and GSK3/β-TrCP, that target NRF2 for ubiquitin/proteasome degradation [20,29]. We used the NRF2 mutants, NRF2^ΔETGE^ and NRF2^6SA^, which respectively escape to KEAP1 or β-TrCP-mediated degradation (see Figure 2C for details). Glioblastoma cells U-87 MG and U-373 MG were transduced with lentiviral vectors expressing wild-type NRF2, NRF2^ΔETGE^, NRF2^6SA^, or empty vector as a control, and then with control interference (shco) or with shWIP lentiviral vectors. After 3 days of WIP interference, we reproduced the drop in the levels of ectopically expressed wild-type NRF2, and the NRF2^6SA^ levels were similarly decreased. By contrast, the NRF2^ΔETGE^ levels were insensitive to the WIP knockdown (Figure 2D,G). These results suggest that the drop in NRF2 caused by WIP silencing is due to the induction of the KEAP1 degradation pathway. To further confirm this observation, the control and WIP-knocked-down cells were treated with the selective KEAP1 inhibitor sulforaphane (SFN) (Figure 2E,H). Both SFN-treated cell lines accumulated NRF2 despite the WIP depletion. The accumulation was lower than in the control cells, but it must be noted that the initial amount of NRF2 in the WIP-silenced cells was also lower. Additionally, we used lentiviral KEAP1 knock-down in combination with WIP knockdown (Figure 2F,I). KEAP1 deficiency increased the NRF2 levels, as expected, and also rescued the NRF2 downregulation elicited by the WIP knockdown. Altogether, these genetic and chemical approaches indicate that WIP protects NRF2 from KEAP1-mediated degradation.

### 3.3. The Control of the Cellular Redox State by WIP Is NRF2-Dependent

**Figure 3:**
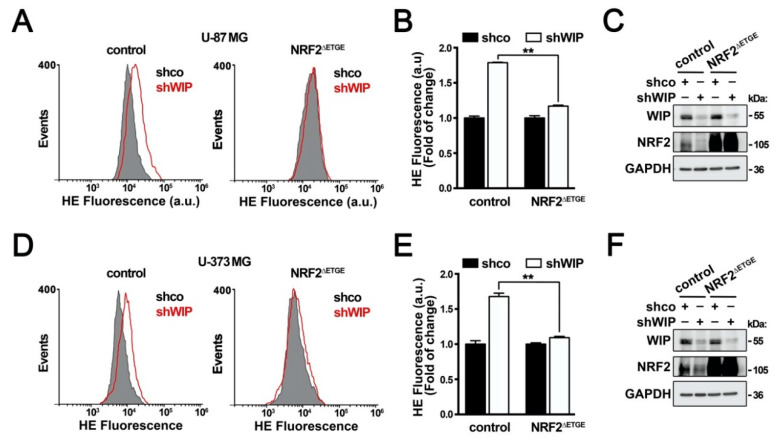
The regulation of redox state by WIP is NRF2-dependent. (**A**–**C**) U-87 MG and (**D**–**F**) U-373 MG glioblastoma cells were transduced with empty lentiviral as control or a lentivirus expressing NRF2^ΔETGE^, and then with a lentivirus expressing shco or shWIP. (**A**,**D**) Changes in intracellular ROS were determined by flow cytometry of HE staining (a.u., arbitrary units of fluorescence emission). A representative sample of 10,000 cells is shown for each condition. (**B**,**E**) Quantification of intracellular ROS production in HE-stained (a.u., arbitrary units of fluorescence emission) (*n* = 3). Data are presented as mean ± S.D. ** *p* ≤ 0.01 according to one-way ANOVA. (**C**,**F**) Representative immunoblots of WIP, NRF2, and GAPDH as a loading control.

To determine if the changes in redox status resulting from WIP depletion are dependent on the observed reduction in NRF2, we analyzed the effect of NRF2^ΔETGE^ in WIP-knocked-down cells. In agreement with Figure 1, WIP silencing increased the intracellular ROS levels, as determined by HE oxidation, but NRF2^ΔETGE^ rescued the normal ROS levels (Figure 3A,B,D,E). Consistently, under these experimental conditions, we confirmed WIP interference and NRF2^ΔETGE^ overexpression by immunoblot (Figure 3C,F). Therefore, WIP requires NRF2 for maintaining the basal redox status.

### 3.4. WIP Does Not Regulate KEAP1 Degradation

**Figure 4:**
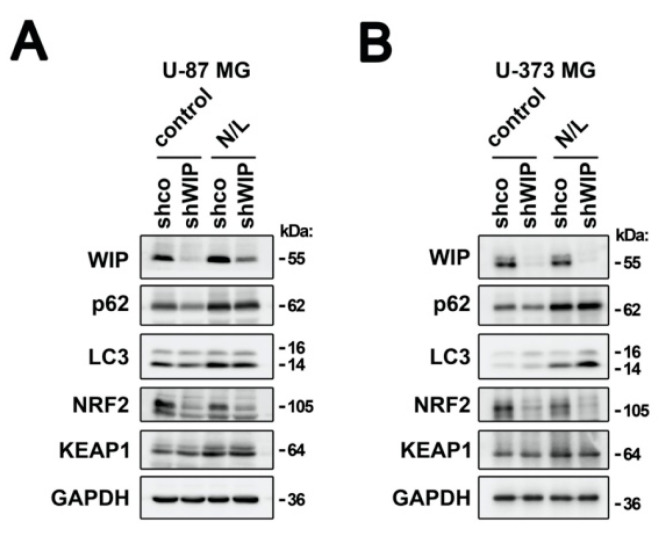
WIP does not regulate KEAP1 degradation. Immunoblots of WIP, p62, LC3, NRF2, KEAP1, and GAPDH as a loading control of U-87 MG (**A**) and U-373 MG (**B**) glioblastoma cells that were transduced with lentiviral vectors containing shco or shWIP and then treated with a standard cocktail of ammonium chloride and leupeptin (N/L), to 20 µM and 100 µM, respectively, during 24 h.

One possible explanation for the protective effect of WIP over the KEAP1-mediated degradation of NRF2 might be that WIP modifies the stability of KEAP1. KEAP1 degradation involves selective autophagy through interaction with the cargo protein p62 [30]. Considering that WIP participates in multiple aspects of endosomal trafficking, we explored if WIP might be controlling KEAP1 degradation through autophagy. WIP-depleted cells were treated with a standard cocktail of ammonium chloride and leupeptin (N/L), which inhibits lysosomal proteases [24,31]. In U-87 MG and U-373 MG glioblastoma cells, the inhibition of autophagy was confirmed by the increased accumulation of lipidated LC3-II and p62 proteins (Figure 4A,B). In agreement with other reports, KEAP1 was also accumulated [30] (Figure 4A,B). Regarding NRF2, its levels decreased when KEAP1 was protected from autophagy by the N/L cocktail, and the WIP-depleted cells still led to the further degradation of NRF2. Even more, as shown in Figure 4A,B, the KEAP1 levels were similar in the control and WIP-knocked-down cells, even in the absence of the autophagy inhibitors, therefore excluding other potential pathways for KEAP1 degradation. Hence, the effect of WIP on KEAP1 inhibition is at least in part independent of its degradation.

### 3.5. WIP-Depleted Cells Exhibit Increased Co-Localization between KEAP1 and F-Actin

The remodeling of the Actin cytoskeleton is a well-established function attributed to WIP. Therefore, we analyzed if WIP-silencing might alter the dynamics of Actin microfilaments in the U-87 MG and U-373 MG glioblastoma lines. Following three days of WIP knockdown, there was not a change in the amount of Actin detected by immunoblot (Supplementary Figure S2a,d), but when the cells were stained with Phalloidin, a toxin that selectively binds to filamentous Actin (F-Actin), WIP depletion led to a ~1.5-fold increase in polymerized Actin over the control (Supplementary Figure S2b,c,e,f).

**Figure 5:**
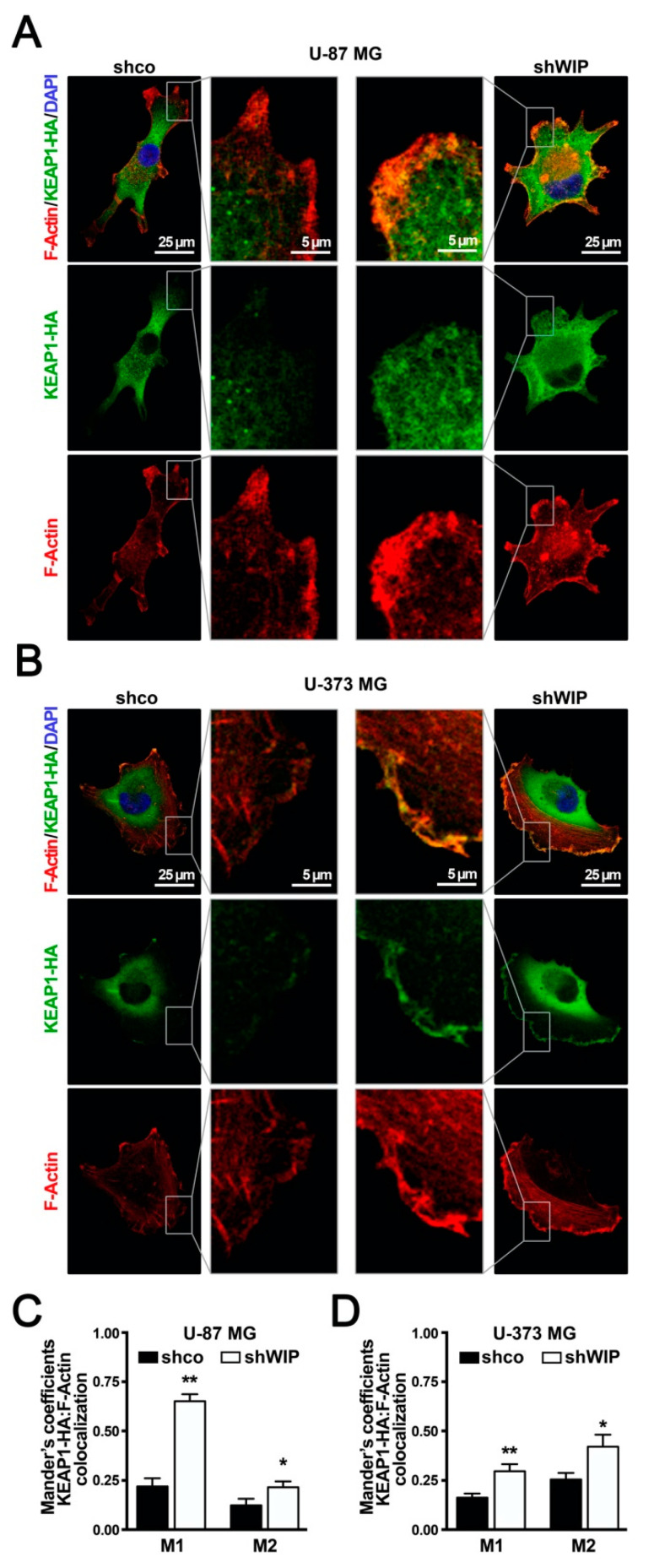
WIP knocked-down cells exhibit increased co-localization between KEAP1 and F-Actin. U-87 MG (**A**,**C**) and U-373 MG (**B**,**D**) glioblastoma cells were transduced with lentivirus expressing KEAP1-HA and then with a lentivirus expressing shco or human shWIP. (**A**,**B**) Immunostaining of KEAP1 with HA antibody and counterstaining with Phalloidin and DAPI to show F-Actin and nuclei, respectively. (**C**,**D**) Quantification of colocalization between F-Actin and KEAP1-HA expressed as Manders’ overlap coefficients. Fluorescence was quantified in 1 µm-thick stacks using the JACoP plugin of ImageJ software. Data are presented as mean ± S.E.M. (*n* = 20) * *p* ≤ 0.05, ** *p* ≤ 0.01 according to one-way ANOVA.

KEAP1 was initially defined as a cytoskeleton-associated protein [32,33]. Therefore, we analyzed if WIP maintains NRF2 stability by altering the KEAP1 interaction with the Actin cytoskeleton. Due to the low KEAP1 levels and lack of suitable antibodies for the immunofluorescence of endogenous KEAP1, the co-localization between KEAP1 and the polymerized Actin was studied in cells ectopically expressing HA-tagged KEAP1. In the U-87 MG and U-373 MG glioblastoma lines, a pool of KEAP1 was localized in at the perinuclear cytoplasm, and another was close to the plasma membrane clustered in the podosomes, invadosomes, or other Actin-rich structures. In WIP-depleted cells, we found increased co-localization between KEAP1-HA and F-Actin at the Actin-rich structures of the plasma membrane (Figure 5A,B) that was further quantified using Manders’ overlap coefficients (Figure 5C,D).

Altogether, all these data indicate that WIP deficiency alters Actin dynamics, possibly increasing the interaction of KEAP1 with microfilaments, increasing the cytoplasmic retention and degradation of NRF2, and altering the cellular basal redox state.

## 4. Discussion

The role of WIP in the organization of the Actin microfilaments endows this protein with a unique function in cytoskeletal remodeling and vesicle trafficking, which are required for cell survival, proliferation, and tumorigenesis [6,10]. In this study, we show for the first time that WIP also participates in the control of redox status by protecting NRF2 from ubiquitin-proteasome degradation. The importance of NRF2 in cancer progression has been widely described as it increases tolerance to the high ROS production that characterizes tumor cell growth [14,34,35,36]. Consistent with our observations in cell culture, glioma patients also exhibit high expression levels of both WIP and NRF2, resulting in poor prognosis [6,37,38].

The best-characterized pathways that control NRF2 stability are KEAP1 and β-TrCP [39]. These two mechanisms appear to obey to different cellular circumstances. Thus, KEAP1 connects NRF2 degradation with redox signaling and β-TrCP with growth factor signaling. The use of specific NRF2 mutants that are resistant to KEAP1 or β-TrCP-mediated degradation as well as the chemical and genetic silencing of KEAP1 allowed us to conclude that WIP restrains the capacity of KEAP1 to target NRF2 for degradation. Therefore, our results favor a role of WIP in the control of oxidative stress.

Considering that WIP participates in vesicular trafficking, we analyzed if the regulation of NRF2 stability by WIP might involve KEAP1 degradation by autophagy. KEAP1 interacts with the autophagy cargo protein p62/SQSTM1, which sequesters the complex in inclusion bodies and promotes its degradation [40,41]. The participation of WIP in autophagy has not been addressed yet, but the Wiskott–Aldrich syndrome protein (WASP), a binding partner of WIP in innate immune cells, appears to participate in autophagy [42]. Thus, WASP deficiency results in impaired autophagic p62/LC3 recruitment and defective formation of canonical autophagosomes [42]. However, although the inhibition of autophagy led to a concomitant increase in KEAP1, this effect was independent of WIP, because WIP depletion did not alter KEAP1 levels at the baseline or following autophagosome inhibition. The most characterized WIP partner in non-immune cell types, including glioblastomas, is neural-WASP ((N)-WASP) [43,44]. WIP reorganizes of the Actin cytoskeleton in glioblastoma cells in a (N)-WASP-dependent and independent manner [5,45,46], and therefore future studies are required to determine if (N)-WASP, alone or together with WIP, might modulate the autophagy flux in these cell types.

Before the realization that KEAP1 is an E3 ligase adaptor to the CUL3/RBX complex [47,48], it was considered that KEAP1 binds to the Actin cytoskeleton and traps NRF2 in the cytoplasm, thereby preventing its nuclear translocation and transcriptional activity [32]. Drugs that disassemble or stabilize the Actin cytoskeleton led to increased or decreased NRF2 levels, respectively [32], and it was suggested that, by analogy with other proteins that contain a Kelch repeat, KEAP1 might bind F-Actin [32]. Although later on it was found that the Kelch repeats are actually a WD40 propeller involved in NRF2 ubiquitination, these studies provided evidence for the binding of KEAP1 to Actin. The association of KEAP1 with Actin-rich adhesion complexes was further analyzed in different tissues and cell types [33]. KEAP1 is present in focal adhesions of kidney proximal tubule cells and in adherens junctions at the base of the bile canaliculi, in association with Actin. KEAP1 appears to play also a role in podosome formation [49]. Our results are in agreement with an interaction between KEAP1 and F-Actin, and show that this association might be responsible for increased ubiquitination of NRF2 in the absence of WIP. However, we must consider that WIP is a cellular multi-tasker that participates in multiple protein-protein interactions, as it has been recently reviewed [50], and therefore, we cannot exclude the possibility that, besides Actin, other proteins interacting with WIP might play a relevant role in KEAP1 repression. These proteins, most of them interacting with the proline-rich central portion of WIP through its SH3 domain, might include Cortactin, Profilin1/2, and NCK1/2, among others [50], and have functions beyond cytoskeletal dynamics that might regulate the KEAP1/NRF2 axis. For instance, *Nck1*-knockdown in adipocyte cell lines leads to their impaired differentiation in response to platelet-derived growth factor α, accompanied by the increased activation of the transcription factor NRF2 [51]. Whether this pathway of NRF2 regulation is connected to WIP/KEAP1 axis remains to be elucidated.

More recently, a new connection between the NRF2 system and the Actin cytoskeleton has been reported to control of NRF2 activity by the Arp2/3 complex [49], although the role of WIP was not analyzed. The Arp2/3 complex catalyzes the polymerization of monomeric Actin into branched Actin microfilaments [52]. It was found that depletion of the Arp2/3 complex by knockout of *Arpc4* leads in keratinocytes to a reduction in Actin microfilaments, including stress fibers, and NRF2 hyperactivation. Our results are in agreement with this study as we found that WIP-depletion leads to the opposite effect—i.e., more stress fibers correlated with a lower NRF2 activity. However, in keratinocytes these authors did not detect the expected increase in the NRF2 protein levels despite the fact that they found the increased phosphorylation of the Ser40 of NRF2. This result is contradictory, as Ser40 is located at the Neh2 domain of NRF2 that binds to KEAP1 and has been reported as a mechanism to escape KEAP1-targeted degradation [49]. Further studies will be required to determine if indeed the hyperactivation of NRF2 is or not related to impaired KEAP1 activity in keratinocytes.

There is abundant evidence of the effect of ROS in Actin remodeling. For instance, several critical cysteine and methionine amino acids are susceptible to oxidation in Actin, resulting in alterations in Actin polymerization [53]. Thus, NOX-derived ROS promote the formation Actin-rich protrusions of the plasma membrane [54] and mitochondrial ROS support KRAS-induced anchorage-independent growth of murine lung cancer cells [55]. Therefore, we propose that WIP might act as a regulator of the overall redox state, through NRF2 regulation, and provide a feedback mechanism for the protection of Actin microfilaments from uncontrolled oxidation.

## 5. Conclusions

WIP participates in tumor progression and promotes cancer cell survival. Here, we report a new role of this multi-taker protein in the control of the redox adaptation of cancer cells by preventing the KEAP-dependent degradation of NRF2. Most likely this effect is due to the reorganization of the Actin cytoskeleton. Therefore, we propose that WIP might act as a regulator of the overall redox state, through NRF2 regulation, and provide a feedback mechanism for the protection of Actin microfilaments from uncontrolled oxidation. Our study provides a new role of WIP in maintaining the oxidant tolerance of cancer cells, which may have therapeutic implications.

## Supplementary Materials

The following are available online at https://www.mdpi.com/2076-3921/9/9/773/s1: Figure S1: Two different WIP shRNAs decrease NRF2 protein levels. Figure S2: WIP knocked-down cells exhibit increased F-Actin levels. Table S1: Oligonucleotides used for qRT-PCR.
