# Supplementary material for: WIP Modulates Oxidative Stress through NRF2/KEAP1 in Glioblastoma Cells
Source: Antioxidants (Basel). 2020 Aug 20;9(9):773. doi: 10.3390/antiox9090773 (PMC7555221; doi:10.3390/antiox9090773)

WIP modulates oxidative stress through NRF2 / KEAP1 in glioblastoma cells

Maribel Escoll , Diego Lastra, Natalia Robledinos-Antón, Francisco Wandosell,

Inés María Antón and Antonio Cuadrado

Supplementary Table S1. Oligonucleotides used for qRT-PCR.

Gene	Forward Sequence 5´-3´	Reverse Sequence 5´-3´
HMOX1	TGCTCAACATCCAGCTCTTTGA	GCAGAATCTTGCACTTTGTTGC
NQO1	GTTCATAGGAGAGTTTGCTT	CCTTGCAGAGAGTACATGGA
GCLM	TCAAACTCTTCATCATCAAC	TTCTAATTCCTCCCAGTAAG
GAPDH	CTCTCTGCTCCTCCTGTTCGAC	TGAGCGATGTGGCTCGGCT
TBP	TGCACAGGAGCCAAGAGTGAA	CACATCACAGCTCCCCACCA

Supplementary Figure S1. Two different WIP shRNAs decrease NRF2 protein levels. U-373 MG and U-87 MG glioblastoma cells were transduced with lentiviral vectors containing shcontrol (shco) or with two different human shWIP, shWIP-1 (also called shWIP in the rest of the figures) or shWIP-2. Representative immunoblots of WIP, NRF2 and GAPDH as a loading control (n = 3).

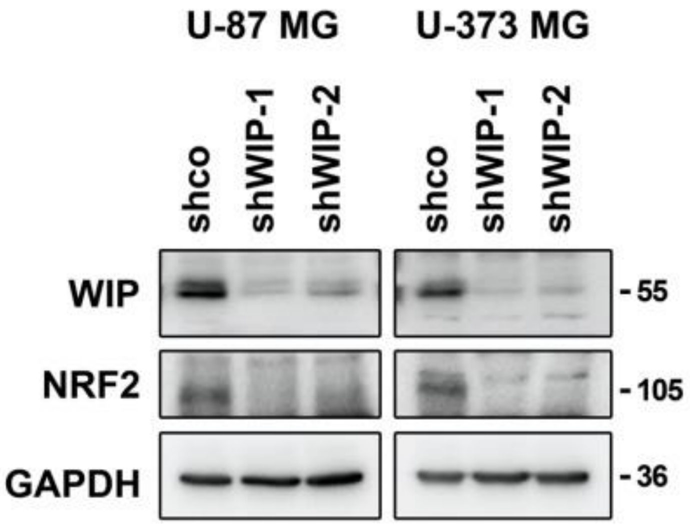

Supplementary Figure S2: WIP knocked-down cells exhibit increased F-Actin levels. U-87 MG **(A, B, C)** and U-373 MG **(D, E, F)** glioblastoma cells were transduced with lentiviral vectors containing shco or human shWIP. (**A, D**) immunoblots showing Actin levels in control vs. shWIP cells. (**B, E**) F-Actin was stained with Phalloidin and nuclei were counterstained with DAPI. (**D, F**) Quantification of fluorescence intensity. Data are presented as mean ± S.E.M. (n = 100). * $p \leq 0.05$, ** $p \leq 0.01$ according to a Student's t-test.

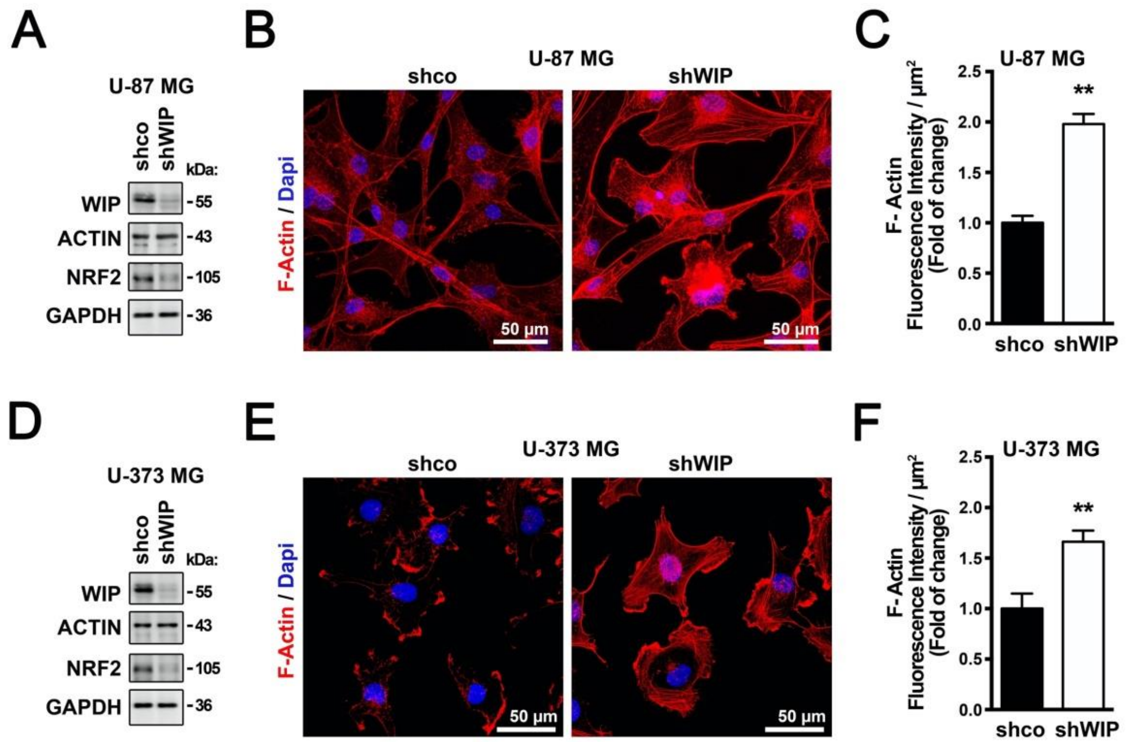

Supplement: Supplementary file 1 [file antioxidants-09-00773-s001.pdf]
